# Editorial: Synthesis of Novel Hydrogels With Unique Mechanical Properties

**DOI:** 10.3389/fchem.2020.595392

**Published:** 2020-10-20

**Authors:** Ying Li, Kerstin G. Blank, Fei Sun, Yi Cao

**Affiliations:** ^1^Institute of Advanced Materials and Flexible Electronics (IAMFE), School of Chemistry and Materials Science, Nanjing University of Information Science and Technology, Nanjing, China; ^2^Mechano(bio)chemistry, Max Planck Institute of Colloids and Interfaces, Potsdam, Germany; ^3^Department of Chemical and Biological Engineering, The Hong Kong University of Science and Technology, Kowloon, China; ^4^National Laboratory of Solid State Microstructures, Department of Physics, Nanjing University, Nanjing, China; ^5^Institute for Brain Sciences, Nanjing University, Nanjing, China; ^6^Chemistry and Biomedicine Innovation Center, Nanjing University, Nanjing, China; ^7^Shenzhen Research Institute of Nanjing University, Shenzhen, China

**Keywords:** hydrogel, mechanical property, network topology, crosslinking, biomaterials

Hydrogels are wet polymeric or supramolecular networks showing properties of both solid and liquid. They are promising materials for a broad range of applications, including cell culturing (Caliari and Burdick, 2016), tissue engineering (Vedadghavami et al., 2017), wound dressing (Ghobril and Grinstaff, 2015), controlled drug release (Li and Mooney, 2016), flexible electronics (Yang and Suo, 2018), and soft robotics (Liu et al., 2020), just to name a few. In many of these applications, the mechanical properties of hydrogels play an important role and have gathered increasing attention. However, despite considerable progress and increasing numbers of papers published in recent years, it remains challenging to formulate hydrogels with controlled mechanical properties to meet diverse applications.

To provide a forum to discuss the latest researches in this field, we created a Research Topic on “Synthesis of Novel Hydrogels with Unique Mechanical Properties.” In this Research Topic, we have published 13 papers, including 12 research articles, and one review. These papers highlight several emerging trends in this direction.

First, biomacromolecules are widely used as the building blocks to modulate the mechanical properties of hydrogels (Li et al., 2020). For example, Xiang et al. showed that photo-hardening and photo-weakening hydrogels can be engineered using a visible light-cleavable fluorescent protein, PhoCl, designed by the Campbell group (Zhang et al., 2017). The mechanical properties of the hydrogels are altered based on photo-induced bond rupture and the subsequent unfolding of the proteins. Lu et al. reported that hydrogels with high mechanical strength can be prepared by incorporating albumin in the hydrogel network. They introduced a heat-processing approach to partially unfold and aggregate albumin, which added abundant physical crosslinks to the chemically crosslinked hydrogels. The hybrid crosslinking mechanism is responsible for the high compressive and tensile strength of the resulting hydrogels. Another interesting example is the work by Cui et al. It is shown that hybrid hydrogels made of DNA and synthetic polymers can serve as recyclable gene carriers for cell-free protein synthesis. To achieve optimal production yield, the hydrogels should be neither too soft nor too hard. Soft hydrogels are easily damaged by the shaking forces during protein production. On the other hand, hard hydrogels exhibited dense network structures, which slowed down the diffusion of the reactants and lowered the efficiency of protein production.

Second, crosslinking chemistry is emphasized for hydrogel preparation. To incorporate new building blocks, such as proteins, novel hydrogel-crosslinking methods have been developed. Camp et al. reported an optimized ruthenium-catalyzed photo-crosslinking strategy to trigger the formation of dityrosine in artificial proteins in a controllable fashion, yielding hydrogels of predictable mechanical strength. Using the same catalyst, Farajollahi et al. showed that the ruthenium-mediated photoreaction led to unexpected disulfide metathesis in minicollagen. The obtained hydrogels were crosslinked by redox sensitive disulfide bonds and their mechanical properties were modulable and reconfigurable. In parallel to the photo-chemistry approach, enzyme mediated crosslinking is also biocompatible and controllable, making the resulting hydrogels suitable for biomedical applications. Shen et al. reported a novel dual enzyme strategy to fabricate hydrogels of tunable strength. They employed a dual enzyme system, HRP@GOx, with distinct catalytic activities for orthogonal reactions. In a fast reaction, tyrosine-modified chondroitin sulfate was crosslinked to form the first network. In a slow polymerization of various monomers, a second network was formed. Temporally decoupling the two-reaction kinetics allowed the hydrogel to be easily fabricated using 3D printing while further polymerization allowed for tuning the mechanical properties. This new technique is expected to find broad biomedical applications.

Third, it is increasingly realized that hydrogel network topology, other than the specific crosslinking chemistry, is important for the mechanical properties of hydrogels. Grad et al. showed that the same crosslink can lead to distinct viscoelastic behaviors, depending on the structure and organization of the polymer to be crosslinked. A self-assembled semi-flexible fibrous network displayed a relaxation time almost two orders of magnitude slower than a flexible random-coil network of four-armed polyethylene glycol. It is proposed that the distinct stress-relaxation behaviors of the two systems are determined by their different network topologies. In the random-coil network, crosslink dissociation causes immediate relaxation while in the fibrous network multiple bonds need to dissociate to allow network relaxation. This shows that it is possible to decouple the microscopic and macroscopic dynamics of hydrogels using different network topology.

Fourth, computational studies can provide tremendous insights. Using molecular dynamics simulations, Zidek et al. investigated hydrogels made of linear chain connected micelles. It was shown that the micelles transform into fibrils upon deformation and that such evolution largely depends on strain rates. Their model also suggested a set of conditions in which the micelle-to-fibril transition is favorable, which may inspire future experimental studies. Moreover, simulations using mesoscopic models can bridge the conformational change of polymer chains at the microscopic level to the deformation of hydrogel at the macroscopic level. To this end, Lei et al. constructed coarse-grained bead-spring models for hydrogels made of a single network of polyacrylamide. They studied the strain and fracture behavior using dissipative particle dynamics. It is shown that the simulations adequately replicate the hyperelasticity and the viscoelasticity of the hydrogels observed experimentally. It was further possible to predict the fracture stretch, based on the fracture stretch of C-C bonds. It is interesting to see whether this model can be used to also predict the mechanical behavior of more complex network structures.

Last but not least, the connection of diverse mechanical properties with specific applications has been exemplified in numerous systems. Malik et al. optimized chitosan/xanthan gum hybrid hydrogels for antiviral drug delivery. Kong et al. reported the synthesis of alginate fibroid hydrogels crosslinked by dynamic ionic interactions. These hydrogels were suitable for the removal of heavy metal ions in water. Zhang et al. demonstrated that metal ion crosslinked organohydrogels can be fabricated in water-cryoprotectant binary solvent. The hydrogels exhibited high toughness and function properly at extreme temperatures as low as −20°C and −45°C. They are promising for applications in harsh temperature conditions. The mechanical properties of hydrogels are obviously important for tissue engineering. Bao et al. summarized the recent development of hydrogels made of natural polymers for cartilage repair and regeneration. Clearly, the mechanical properties of the hydrogels played a pivotal role in this direction.

In summary, this Research Topic highlights the importance of mechanical properties for the development of specific hydrogel applications and provides experimental methods and theoretical frameworks to design hydrogels with controllable and predictable mechanical properties.

## Author Contributions

All authors listed have made a substantial, direct and intellectual contribution to the work, and approved it for publication.

## Conflict of Interest

The authors declare that the research was conducted in the absence of any commercial or financial relationships that could be construed as a potential conflict of interest.
